# Time-dependent synergism of five-component mixture systems of aminoglycoside antibiotics to *Vibrio qinghaiensis* sp.-Q67 induced by a key component†

**DOI:** 10.1039/d0ra00915f

**Published:** 2020-03-26

**Authors:** Jin Zhang, Meng-ting Tao, Chongchong Song, Bai Sun

**Affiliations:** Key Laboratory of Water Pollution Control and Wastewater Resource of Anhui Province, College of Environment and Energy Engineering, Anhui Jianzhu University Hefei 230601 PR China ginnzy@163.com +86-180-1958-0589

## Abstract

A large number of antibiotics are entering the aquatic environment accompanying human and animal excreta, which will threaten the survival of aquatic organisms and even human health. It has been found that binary mixtures of aminoglycoside (AG) exhibit additive action and can be evaluated well by a classical model, concentration addition (CA) in our past study. Therefore, to investigate the toxicity interaction within multi-component mixtures of AG antibiotics, five antibiotics, kanamycin sulfate (KAN), neomycin sulfate (NEO), tobramycin (TOB), streptomycin sulfate (STS), and gentamicin sulfate (GEN), were selected to construct five-component mixture systems by a uniform design ray method. The toxic effects (luminescence inhibition) of single antibiotic and five-antibiotic mixture systems towards a photobacterium *Vibrio qinghaiensis* sp.-Q67 (*V. qinghaiensis*) in different exposure time (0.25, 2, 4, 8, and 12 h) were determined by the time-dependent microplate toxicity analysis method. The concentration-effect data were fitted by a nonlinear least square method, toxicity interaction within mixture systems was analyzed by a CA model, and the interaction intensity was characterized by deviation from the CA model (dCA). Besides, the toxicity mechanism of five antibiotics and their five-component mixtures to *V. qinghaiensis* was analyzed by electron microscopy. The results show that toxicity of five antibiotics and their five-component mixture systems to *V. qinghaiensis* is time-dependent and has strong long-term toxicity. Different from binary AG antibiotic mixture systems, five-antibiotic mixture systems exhibit obviously time-dependent synergism. In addition, toxicity of the five-antibiotic mixtures can be 1.4 times higher than that of the mixtures without synergisms at the same concentration level. According to dCA, synergism intensity (dCA) curves of rays move slowly from the high concentration region to the medium or lower one and the maximum dCA values also increase, decrease, or first increase, then decrease with the lengthening of exposure time. The inhibition activity and synergism intensity of mixture rays have good correlation with the concentration ratios of STS, the key component for synergism. The cell morphology of *V. qinghaiensis* indicates the strong toxicity of five antibiotics and their mixture rays is not due to the destruction of cell structure, but the inhibition of the light-emitting activity of the photobacterium.

## Abstract 2

There was time-dependent synergism in the five-component mixture systems of five antibiotics, which induced by STS.

## Graphical abstract

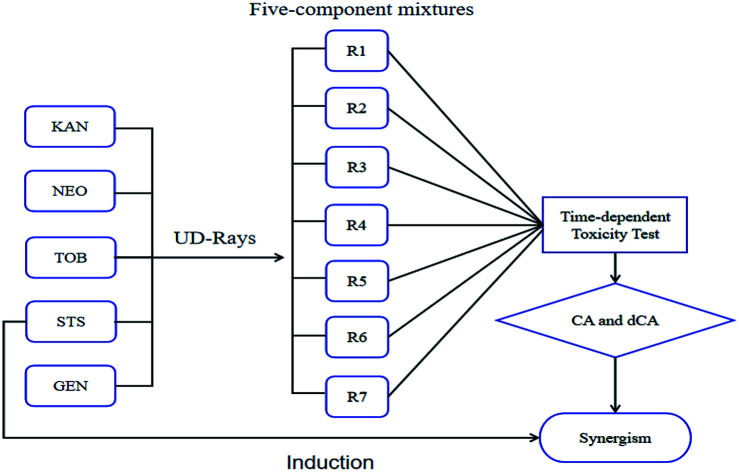

## Introduction

Aminoglycoside (AG) antibiotics are important drugs in the treatment of severe aerobic Gram-negative bacilli infection. However, only a small part of these drugs are absorbed by the body after ingestion, and most of them are discharged into the water environment through the feces and urine of patients and poultry, thus posing a threat to the survival of aquatic organisms and even the health of humans.^1–4^

Exposure time and interaction, synergism and antagonism, between mixture components may increase the toxicity of pollutants at a certain concentration.^5–10^ Zhang *et al.*^11^ found that four AG antibiotics and their mixtures，ampumycin sulfate, dihydrostreptomycin, kanamycin sulfate and neomycin salt, have obvious time-dependent toxicity to *Vibrio qinghaiensis* sp.-Q67 (*V. qinghaiensis*). Liu *et al.*^12^ found that the binary mixture of imidacloprid and pirimicarb showed synergism. Tang *et al.*^13^ studied the combined toxicity of ternary mixtures of heavy metals, ionic liquids and pesticides, and found that there was obvious synergism in the ternary mixture system. Therefore, the effects of exposure time and interaction between mixture components should be considered in order to accurately assess the environmental risk of AG antibiotics.

Usually, analysis of toxicity interaction within mixtures is relative to a standard reference model, so it is very important to select a suitable reference model when evaluating the toxicity (CA) is considered to be a relatively conservative model for predicting mixture toxicity^14^ and widely used by many scholars.^15–18^ However, CA model cannot quantitatively evaluate the degree of synergism or antagonism. Deviation from CA model (dCA) can visualize the difference between experimental observation and model prediction. The greater the absolute value of dCA is, the greater the degree of interaction between the components of the mixture system.

Some studies found that toxicity and toxicity interaction of complex mixture system may be closely related to a key component.^19^ Fan *et al.*^20^ found that polymyxin B sulfate may be the key component of antagonism between ionic liquids and antibiotic. Zhang *et al.*^21^ found that 1-butyl-3-methylimidazolium-octyl sulfate could induce antagonism in quaternary ionic liquid mixtures. So whether there are key components in AG antibiotics mixture system is also the concern of this paper.

As stated above, this study aims to investigate toxicity interaction, synergism or antagonism, within multi-component AG antibiotic mixtures. To do so, five AG antibiotics, kanamycin sulfate (KAN), neomycin sulfate (NEO), tobramycin (TOB), streptomycin sulfate (STS), gentamicin sulfate (GEN), were selected as the research objects, and *V. qinghaiensis* as the tested organisms to explore the toxicity characteristics of AG antibiotics and their mixtures. The five-component mixture systems of the five antibiotics were constructed by uniform design method.^22–24^ The inhibition data of single antibiotic and its five-component mixtures to *V. qinghaiensis* at different exposure time (0.25, 2, 4, 8, and 12 h) were determined by time-dependent microplate toxicity analysis method.^25–27^ The concentration-effect data were fitted by nonlinear least square method, the toxicity interaction of mixture systems was analysed by concentration addition model (CA) with 95% confidence interval,^17–22,28^ and the degree of toxicity interaction was characterized by deviation from CA model (dCA). Besides, the luminescence inhibition mechanism of five antibiotics and their five-component mixtures to *V. qinghaiensis* was preliminarily determined by observing the cell morphology.^29,30^ The experimental data obtained would provide a reference for the assessment of the ecological risk of the AG antibiotics.

## Experimental

### Chemicals

Five antibiotics, kanamycin sulfate (KAN), neomycin sulfate (NEO), tobramycin (TOB), streptomycin sulfate (STS), gentamicin sulfate (GEN) are purchased from Shanghai Field Biotechnology Co. Ltd. Some physical properties of the chemicals are listed in Table 1. The storage solution was prepared with Milli-Q water and stored at 4 °C.

**Table 1:** Some physical properties, stock concentration and dilution factor (*f*) of five antibiotics

Name	Abbr.	MW	CAS RN	Stock (mol L^−1^)	*f*
Kanamycin sulfate	KAN	582.58	25389-94-0	1.10 × 10^−5^	0.68
Neomycin sulfate	NEO	908.880	1405-10-3	1.12 × 10^−5^	0.68
Tobramycin	TOB	467.514	32986.56-4	2.19 × 10^−6^	0.68
Streptomycin sulfate	STS	1457.38	3810-47-0	6.56 × 10^−7^	0.68
Gentamicin sulfate	GEN	575.67	1405-41-0	3.44 × 10^−5^	0.68

### Bacterial culture

The freeze-dried luminescent bacterium *V. qinghaiensis* was purchased from Beijing Hamamatsu Corp., Ltd. (Beijing, China). The preparation of the culture medium and culture process of *V. qinghaiensis* were detailed in the literature.^25–27^

### Time-dependent toxicity test

The determination process of luminescence inhibition of single antibiotic and its five-component mixture systems to luminous bacteria referred to the relevant literature.^11,19^ Using 96 microporous plate as the experimental carrier, 200 μL Milli-Q water was added to the surrounding 36 holes to prevent the edge effect. In the 2nd, 6th, 7th and 11th rows of 24 holes, 100 μL Milli-Q water was added as the blank control, the remaining 36 wells were treated and the drug was diluted to 12 different concentration gradients into the 36 wells by a pre-determined dilution factor, and 3 parallel to each concentration gradient. Finally, a volume of 100 μL of a bacterial liquid was added to the holes of the blank hole and the treatment holes, so that the total volume of each hole was 200 μL. Repeat the above operation 3 times, a total of 3 boards. The three plates were cultured in 22 ± 1 °C biochemical incubator. When the exposure time was 0.25 h, 2 h, 4 h, 8 h, 12 h, the relative luminous value (RLU) of each hole was measured by Power Wave microplate spectrophotometer (American BIO-TEK Company). The luminous inhibition rate (*x*) of *V. qinghaiensis* was calculated according to the average RLU value of blank control (*I*_0_) and the average RLU value of each concentration gradient (*I*). The calculation formula was as follows:1
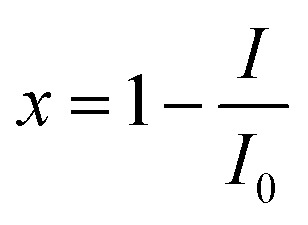


### Five-component mixture design

In order to effectively evaluate synergism or antagonism within complex mixture system, five-component mixture systems of five AG antibiotics was constructed by uniform design method,^22–24^ with a total of seven rays. The basic concentration compositions (BCCs) of seven mixture rays (R1, R2,…, R7) are selected by uniform table of U_7_(7^5^), where the subscript 7 refers to the number of mixture rays, 7 to the number of concentration levels (EC_5_, EC_10_, EC_20_, EC_30_, EC_40_, EC_50_, EC_60_) of various components, and the superscript 5 to the factor/component number. Based on the BCCs, the mixture ratios of components in various mixture rays can be calculated, the calculation results are shown in Table 2. Then, according to the dilution factor obtained from the pre-experiment, 12 fixed concentration ratio points are designed on each ray.^22^

**Table 2:** Component concentration ratios of five-component mixture rays (*P*_i_)

Ray	*P*_KAN_	*P*_NEO_	*P*_TOB_	*P*_STS_	*P*_GEN_
R1	1.70 × 10^−2^	1.21 × 10^−1^	1.60 × 10^−2^	9.00 × 10^−3^	8.37 × 10^−1^
R2	3.30 × 10^−2^	3.44 × 10^−1^	4.60 × 10^−2^	1.00 × 10^−3^	5.76 × 10^−1^
R3	6.50 × 10^−2^	5.59 × 10^−1^	6.00 × 10^−3^	1.10 × 10^−2^	3.60 × 10^−1^
R4	2.53 × 10^−1^	1.16 × 10^−1^	7.00 × 10^−2^	4.00 × 10^−3^	5.57 × 10^−1^
R5	3.22 × 10^−1^	4.00 × 10^−1^	6.00 × 10^−3^	3.00 × 10^−2^	2.42 × 10^−1^
R6	2.99 × 10^−1^	5.83 × 10^−1^	2.80 × 10^−2^	6.00 × 10^−3^	8.40 × 10^−2^
R7	1.49 × 10^−1^	3.72 × 10^−1^	2.70 × 10^−2^	1.00 × 10^−2^	4.41 × 10^−1^

### CRC fitting and effective concentration calculation

The APTox program was used to fit concentration–response curves (CRCs), calculate the median effective concentrations (EC_50_) and the 95% confidence intervals in different exposure time.^22^ The specific fitting functions are Logit (2) and Weibull (3), the expressions are as follows:^22,28^

2 *E* = 1/(1 + exp(−*α* − *β* × log_10_(*c*)))

3 *E* = 1 − exp(−exp(*α* + *β* × log_10_(*c*)))

where *E* represents the effect (0 ≤ *E* ≤ 1), *c* represents the concentration of a single compound or mixture, *α* and *β* are model parameters.

### Identification of toxicological interaction

In this study, the CA model was selected as a reference model to analyse synergism or antagonism within mixture systems. When the CA prediction curve is above, between or below the whole confidence intervals of experimental data, the mixture toxicity is antagonism, additive or synergism, respectively. Mathematically, CA model can be formulated as:^17–22^4
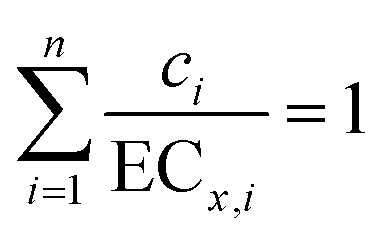
where *n* is the number of mixture components, EC_*x,i*_ the concentration of the *i*th component that provokes *x*% effect when applied individually, and *c*_*i*_ the concentration of the *i*th component in the mixture.

To further characterize the degree of toxicity interaction, dCA was introduced as the evaluation criteria. Mathematically, dCA can be formulated as:

5 dCA = |*E*_OBS_ − *E*_PRD,CA_|

where *E*_OBS_ is the experimental observation effect, and *E*_PRD,CA_ is the prediction effects of CA model at the same concentration. The greater the absolute value of dCA is, the stronger the synergism or antagonism in the mixture.

### The toxicity mechanism of five antibiotics and their five-component mixtures to *V. qinghaiensis*

In order to study the toxicity mechanism of five antibiotics and its five-component mixture to *V. qinghaiensis*, cell morphology of *V. qinghaiensis* exposed to five antibiotics and five-component mixtures with the concentration of EC_50_ at 12 h were observed by electron microscope. The concrete steps are as follows:

*V. qinghaiensis* bacterial suspension during logarithmic growth period was placed in a series of conical flasks. Then, five AG antibiotics and their five-component mixtures were added to the conical flasks with *V. qinghaiensis* bacterial suspension, respectively. The final liquid volume of conical flask was 40 mL. In each conical, the concentrations of each antibiotics and mixtures in the suspension were equal to the EC_50_ of each antibiotic and their five-component mixtures in 12 h. At the same time, Milli-Q water was added as blank control. All the conical flasks were cultured in a constant temperature incubator at 22 ± 1 °C for 12 h. After 12 h, the cell morphology of *V. qinghaiensis* in bacterial suspension of each conical flask was observed by electron microscope. The specific steps for the preparation of electron microscope samples refer to the relevant literatures.^29,30^

## Results and discussion

### Time-dependent toxicity of single antibiotics

**Fig. 1:**
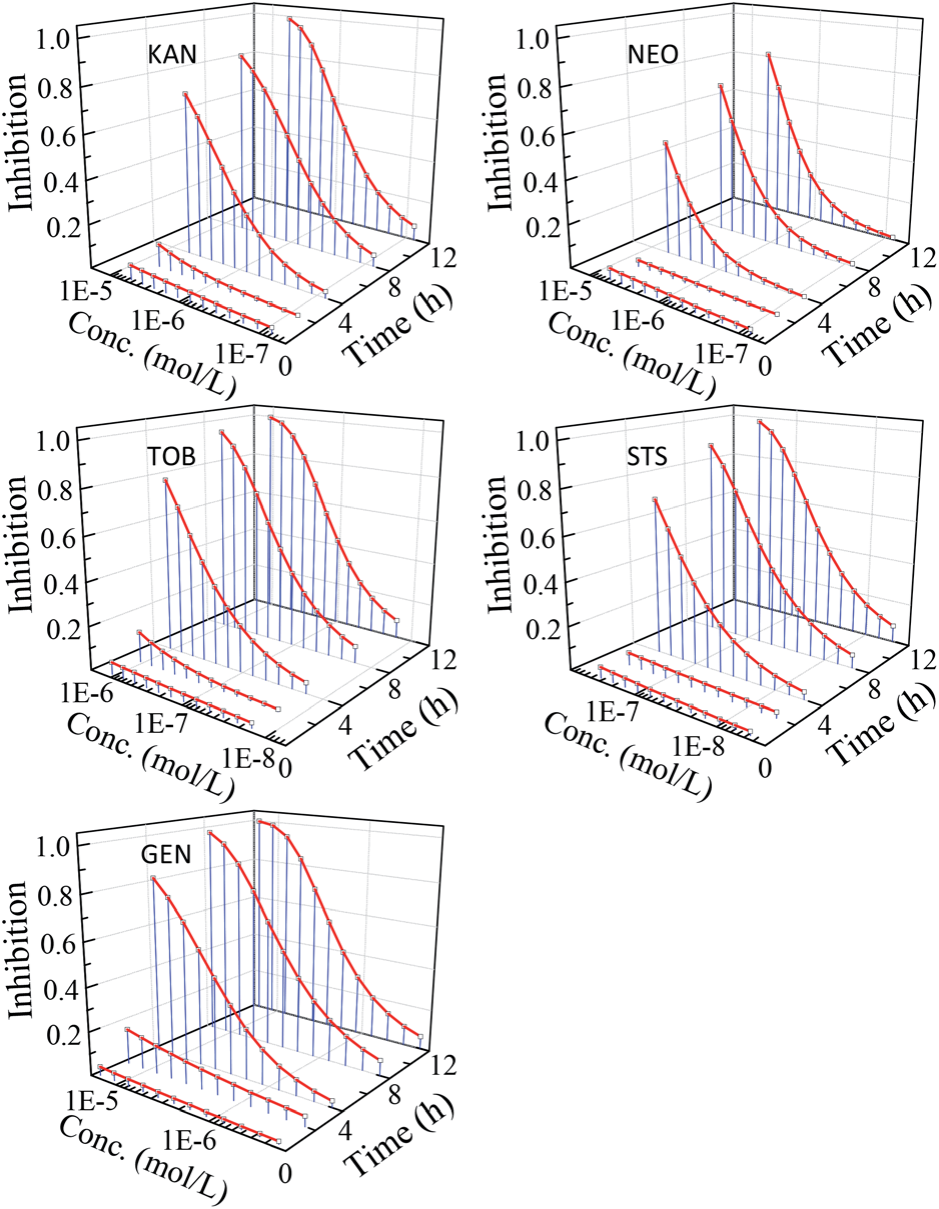
The *t*–CRCs of five antibiotics.

The nonlinear least square method was used to fit concentration-effect data of antibiotics to *V. qinghaiensis*. The results show that the concentration–response relationship of the five AG antibiotics can be effectively characterized by Logit and Weibull functions. The fitted results and some statistics are shown in Table S1.† The time–concentration–response curves (*t*–CRCs) of five antibiotics are shown in Fig. 1.

From Fig. 1, five AG antibiotics showed strong time-dependent inhibition activity to *V. qinghaiensis*, and the time characteristics of the CRCs of five antibiotics were similar. Within 2 h, the luminescence inhibition rates of the five antibiotics were very small, and their *t*–CRCs were almost straight lines, but the inhibition rates increased greatly with the further extension of the exposure time, and the *t*–CRCs became the standard S-shaped. The inhibition activity of the five antibiotics was the highest when the exposure time was 12 h, and the fastest increase rate of inhibition activity was within 2–4 h. This indicates that the five AG antibiotics with the level of experimental concentrations have no obviously short-term toxicity but strong long-term toxicity. Therefore, the effects of exposure time cannot be ignored in the environmental risk assessment antibiotics.^5,7,8^

### Toxicity of five-component mixtures to *V. qinghaiensis*

The concentration-effect data of five-component mixtures at different exposure points can be effectively fitted by Logit and Weibull functions. The fitted results and some statistics are shown in Table S2.† The *t*–CRCs of five-component mixtures are shown in Fig. S1.†

From Fig. S1,† CRCs of seven rays in the five-component mixture system was also time-dependent to *V. qinghaiensis*, the inhibition activity increases with the prolongation of the exposure time, and the time characteristics of CRCs of seven mixture rays were similar to those of a single antibiotic.

**Fig. 2:**
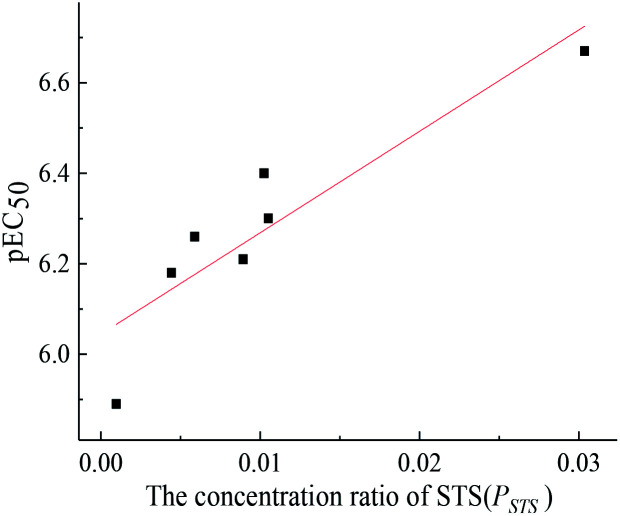
The relationship between pEC_50_ values of seven mixture rays at 12 h and the concentration ratio of STS.

The toxicity of a mixture may be related to some component's concentration ratios.^31,32^ In this study, pEC_50_ (−lg EC_50_) values of seven mixture rays at 12 h were linearly fitted with the concentration ratios of each component. The results showed that there was a good linear relationship between pEC_50_ values of seven mixture rays at 12 h and the concentration ratio of STS (Fig. 2), which indicates that the long-term toxicity of each mixture ray depends on the concentration ratios of STS in the mixture system, and the long-term toxicity of the five-component mixture system can be predicted by using this linear function relationship (pEC_50_ = 22.42*P*_STS_ + 6.04, *R* = 0.9060).

### The time-dependent synergism of five-component mixtures

**Fig. 3:**
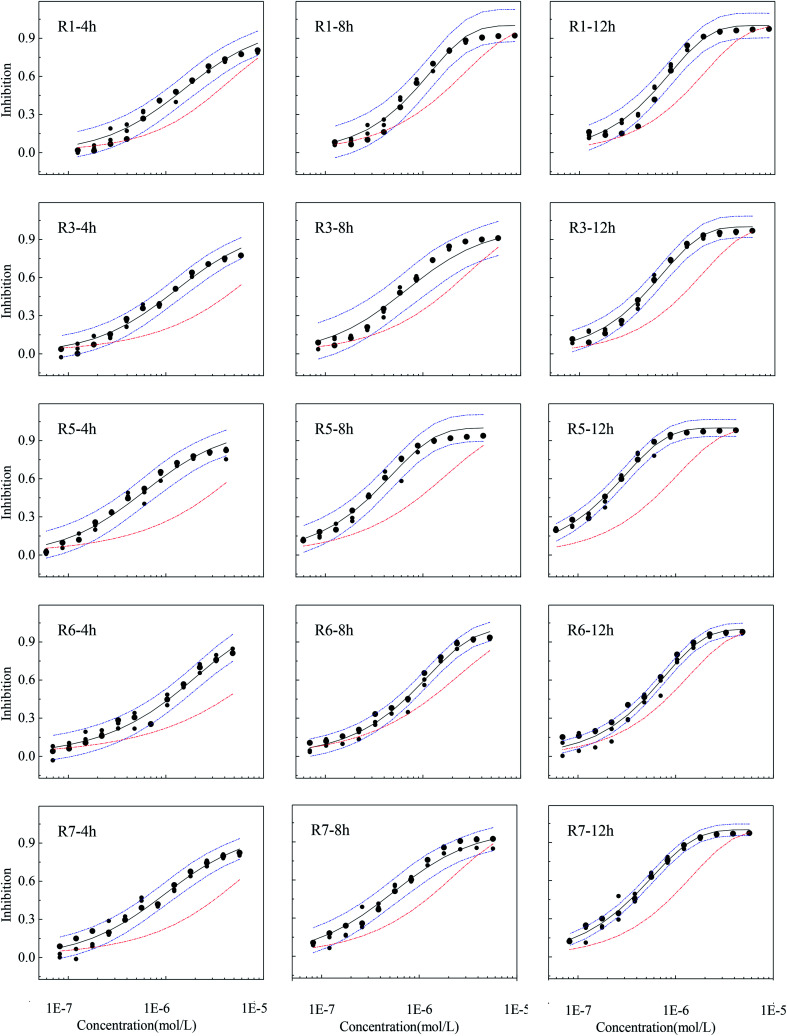
The observed concentration-effect data with 95% confidential intervals, fitted CRCs and predicted curves by CA of representative rays with obvious synergism (●: observed data; —: fitted curve; -·-: predicted curve by CA; -·-: 95% confidence interval).

The results predicted by CA are not always in agreement with the experimental results, and when the CA prediction curve is higher or lower than the confidence interval, there is interaction, synergism or antagonism, within a mixture.^17–22^ The experimental observations of mixture rays with obvious synergism at some exposure time points and their 95% confidence interval, fitting curves and CA prediction results are shown in Fig. 3 (of the rest of CRCs exhibiting additive action are given in Fig. S2†).

From Fig. 3, CA prediction curves located below the confidence intervals of experimental observations from 4 to 12 h, and five out of seven rays in five-component mixture systems showed obvious time-dependent synergism. R2 and R4 had no obvious synergism in the whole exposure time and exhibit classical additive action (Fig. S2†). From Fig. 1 and S2,† the five antibiotics and their five-component mixtures have no obvious toxicity within 2 h probably due the experimental concentrations are well below the acute concentration range. In addition, *V. qinghaiensis* grows very slow in the first stage of exposure time (0.25 h and 2 h). Therefore, the five-component mixtures don't have a synergism at the early stage of exposure (0.25 h and 2 h). It was also noticed that the five rays are time-dependent synergism while R2 and R4 rays with additive action, though the seven rays have the same components. So, the reason mixture rays with the same component exhibit different toxicity interaction are mainly due to effects of the concentration ratios.^32,33^

### The characterization of time-dependent synergism by dCA

**Fig. 4:**
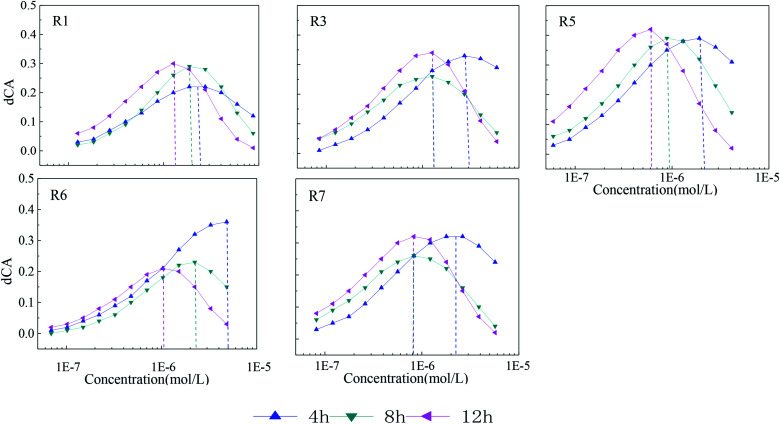
The dCA curves of each mixture ray with obvious synergism in different exposure times.

To further characterize the degree of toxicity interaction, dCA values of each mixture rays with obvious synergism in different exposure times are plotted in Fig. 4.

It can be seen from Fig. 4 that the synergism intensity of the five-component mixture rays with different concentration ratios varies with lengthening of exposure time. From 4 to 12 h, dCA curves of rays move slowly from high concentration region to the medium or lower one, which is probably due to the enough contact between mixture components and bacteria therein. The maximum dCA values also vary with time. From 4 to 12 h, the maximum dCA value of R1 increases gradually with time lengthening, while that of R6 gradually reduces. As for R3, R5 and R7, the maximum dCA value first decrease, then increase. Among the five rays in Fig. 4, R5 shows the strongest synergism with the maximum dCA value of 40%, which means that the toxicity R5 can be at least 1.4 times stronger than that of rays without synergisms. The maximum dCA values of the rest of rays in Fig. 4 are higher than 30%.

By comparing the concentration ratios of seven mixture rays in Table 2, it is found that the synergism in the mixture system is probably correlated with STS. The higher the concentration ratio of STS in the mixture system is, the more obvious synergism in the system. The concentration ratios of STS in R2 and R4 are the smallest compared with other rays, and there is no obvious synergism. The concentration ratio order of STS in the seven rays is: R5 > R3 > R7 > R1 > R6 > R4 > R2, and the synergism intensity (dCA value) order of seven rays is also: R5 > R3 > R7 > R1 > R6 > R4 (R2). However, no significant correlations are found between the synergism of five-component mixture systems and the concentration ratios of other four antibiotics, which indicates that STS may be the key component in five-component mixture systems of AG antibiotics and the time-dependent synergism of the five-component mixtures is induced by the component STS.^19–21^

### The toxicity mechanism to *V. qinghaiensis*

**Fig. 5:**
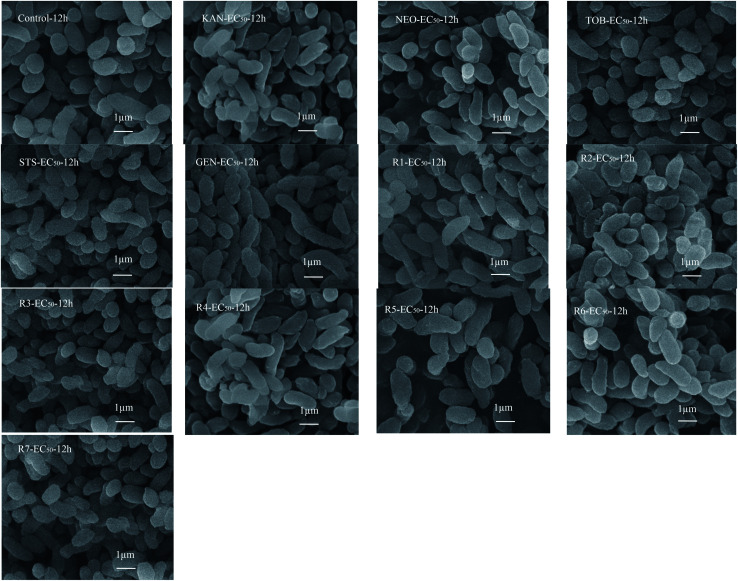
Effects of five antibiotics and their five-component mixture rays on cell morphology of *V. qinghaiensis.* (When the samples were subjected to be scanned by electron microscope, the acceleration voltage and magnification parameters were 10 kV and 15000, respectively).

Toxicity mechanism of AG antibiotics to bacteria is mainly produced by affecting their protein synthesis.^34^ Nastri and Algranati^35^ also demonstrated this by studying the effects of streptomycin and other AG antibiotics on protein synthesis. In this study, cell morphology of *V. qinghaiensis* exposed to five antibiotics and their mixtures with the concentration of EC_50_ were observed by electron microscope to explore the possible toxicity mechanism. The results are shown in Fig. 5.

As can be seen from Fig. 5, most of the cell morphology of *V. qinghaiensis* was maintained in the normal state. Therefore, the toxic effects of the five antibiotics and their five-component mixtures on *V. qinghaiensis* are not caused by destroying cell morphology, but inhibition of luminescence.

## Conclusions

The toxicities of the five antibiotics and their five-component mixture systems to *V. qinghaiensis* are time-dependent and all have strong long-term toxicity. In addition, there is time-dependent synergism in the five-antibiotic mixture systems whose toxicity can be 1.4 times higher than that of the mixtures without synergism. According to dCA, synergism intensity (dCA) curves of rays move slowly from high concentration region to the medium or lower one with the lengthening of exposure time. At the same time, the maximum dCA values also vary (increase, decrease, or first increase, then decrease) with time. The toxicity and synergism intensity of the seven mixture rays have good correlations with the concentration ratios of the key component STS for synergism, and the five antibiotics and their mixture rays can produce strong toxicity to the *V. qinghaiensis* without destroying the cell structure, but inhibiting the light-emitting activity of the photobacterium.

## Conflicts of interest

There are no conflicts to declare.

## Supplementary Material

RA-010-D0RA00915F-s001
